# Impact of 4 weeks of western diet and aerobic exercise training on whole‐body phenotype and skeletal muscle mitochondrial respiration in male and female mice

**DOI:** 10.14814/phy2.15543

**Published:** 2022-12-21

**Authors:** Erin M. McGowan, Sarah E. Ehrlicher, Harrison D. Stierwalt, Matthew M. Robinson, Sean A. Newsom

**Affiliations:** ^1^ School of Biological and Population Health Sciences, College of Public Health and Human Sciences Oregon State University Corvallis Oregon USA

**Keywords:** lipid metabolism, obesity, respirometry, substrate oxidation

## Abstract

High dietary fat intake induces significant whole‐body and skeletal muscle adaptations in mice, including increased capacity for fat oxidation and mitochondrial biogenesis. The impact of a diet that is high in fat and simple sugars (i.e., western diet [WD]), particularly on regulation of skeletal muscle mitochondrial function, is less understood. The purpose of the current study was to determine physiologic adaptations in mitochondrial respiratory capacity in skeletal muscle during short‐term consumption of WD, including if adaptive responses to WD‐feeding are modified by concurrent exercise training or may be sex‐specific. Male and female C57BL/6J mice were randomized to consume low‐fat diet (LFD) or WD for 4 weeks, with some WD‐fed mice also performing concurrent treadmill training (WD + Ex). Group sizes were *n* = 4–7. Whole‐body metabolism was measured using in‐cage assessment of food intake and energy expenditure, DXA body composition analysis and insulin tolerance testing. High‐resolution respirometry of mitochondria isolated from quadriceps muscle was used to determine skeletal muscle mitochondrial respiratory function. Male mice fed WD gained mass (*p* < 0.001), due to increased fat mass (*p* < 0.001), and displayed greater respiratory capacity for both lipid and non‐lipid substrates compared with LFD mice (*p* < 0.05). There was no effect of concurrent treadmill training on maximal respiration (WD + Ex vs. WD). Female mice had non‐significant changes in body mass and composition as a function of the interventions, and no differences in skeletal muscle mitochondrial oxidative capacity. These findings indicate 4 weeks of WD feeding can increase skeletal muscle mitochondrial oxidative capacity among male mice; whereas WD, with or without exercise, had minimal impact on mass gain and skeletal muscle respiratory capacity among female mice. The translational relevance is that mitochondrial adaptation to increases in dietary fat intake that model WD may be related to differences in weight gain among male and female mice.

## Graphical abstract

We and others have established that diets high in fat induce whole‐body and skeletal muscle mitochondrial adaptations; however, the impact of a diet high in both fat and simple sugar (i.e., western diet [WD]) is less understood. The aim of our study was to determine if there are adaptations in mitochondrial respiratory capacity in mice fed WD during a 4‐week intervention, including if such adaptations are further modified by concurrent exercise training or may be sex‐specific. Male mice fed WD displayed significant weight gain and a modest tendency for greater lipid‐supported oxidative phosphorylation whereas female mice did not have any significant differences as a function of the interventions.

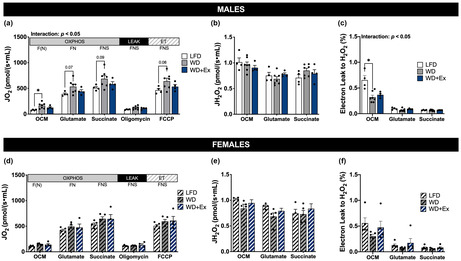

## 1 INTRODUCTION

Skeletal muscle mitochondrial function is linked with metabolic health, whereby metabolic pathologies such as obesity and insulin resistance have been associated with impaired oxidative capacity (Kelley et al., 1999, 2002). Such findings have sparked interest in understanding regulation of skeletal muscle mitochondrial metabolism by diet and obesity. To this end, diets high in fat content induce physiological adaptations to skeletal muscle mitochondria, including greater mitochondrial content (i.e., mitochondrial quantity) and respiratory capacity (Hancock et al., 2008; Leckey et al., 2018; Turner et al., 2007). However, we and others have typically performed such studies using diets high in fat (e.g., 45%–60% of kcal) yet low in simple sugars (Ehrlicher et al., 2020; Hancock et al., 2008; Newsom et al., 2017; Turner et al., 2007). How a diet that is more representative of the obesogenic “western diet” (i.e., high fat, high sucrose, high cholesterol) can impact whole body and skeletal muscle mitochondrial metabolism is less well understood. The high prevalence of obesity highlights the need to understand how diet‐induced weight gain drives changes in skeletal muscle mitochondria. Understanding the impact of a western‐style diet can improve translation of information gained from mouse models to human health.

We previously reported ad libitum consumption of a 60% high‐fat diet for 12 weeks induced greater maximal capacity for mitochondrial fat oxidation and greater rates of mitochondrial protein synthesis compared with a low‐fat diet (LFD; Ehrlicher et al., 2020; Newsom et al., 2017). The 60% high‐fat diet remodeled the mitochondrial proteome toward higher abundance of proteins for fat oxidation (Dasari et al., 2018). There were no changes in respiratory capacity for non‐lipid substrates, indicating lipid‐specific adaptations in skeletal muscle mitochondrial oxidative function (Dasari et al., 2018; Ehrlicher et al., 2020; Newsom et al., 2017). Others have reported increases in skeletal muscle mitochondrial biogenesis and capacity for fat oxidation after only 4 weeks of 50% high‐fat feeding (Hancock et al., 2008). Taken together, diets high in fat content provide a robust stimulus to remodel skeletal muscle mitochondria, with adaptations being specific to enhanced fat oxidation.

Longer‐term western diet (WD) feeding is known to influence skeletal muscle mitochondrial metabolism. For example, Stephenson et al. (2012) reported that male Long‐Evans rats provided WD (17% protein, 43% carbohydrate, 40% fat) for 12 weeks had greater maximal oxidative phosphorylation respiration of non‐lipid substrates compared with rats fed a low fat diet (21% protein, 63% carbohydrate, 16% fat). Rats fed the WD also had greater skeletal muscle activity of β‐hydroxyacyl‐CoA dehydrogenase (β‐HAD) compared with low fat fed rats. When female cynomolgus macaques were provided a WD or Mediterranean‐style diet for 30‐months (both diets only 31% fat), animals fed the WD had greater skeletal muscle capacity for oxidative phosphorylation when respiring lipids, non‐lipid substrates and a combination thereof (Gonzalez‐Armenta et al., 2019). Whether or not such changes in skeletal muscle mitochondrial respiratory function occur during shorter duration WD feeding remains to be tested.

To what extent changes in skeletal muscle mitochondrial metabolism during WD feeding can be modified by exercise and may differ between sexes are also questions of interest. We recently reported that chronic treadmill exercise training increased skeletal muscle mitochondrial respiratory capacity for both lipid and non‐lipid substrates in mice provided 60% kcal from fat (Ehrlicher et al., 2020). The effect of exercise training on skeletal muscle mitochondrial metabolism during WD feeding remains unknown. Furthermore, evidence indicates female mice are protected against diet‐induced obesity (Hwang et al., 2010; Ingvorsen et al., 2017) and exhibit greater mitochondrial capacity for fat oxidation in skeletal muscle compared with males (Cardinale et al., 2018; Miotto et al., 2018; Montero et al., 2018). Identifying potential sex‐based differences in the mitochondrial response to WD, including effect modification by exercise training, is important.

The aim of the current study was to determine if there are physiological adaptations in both lipid and non‐lipid‐supported mitochondrial respiratory capacity in skeletal muscle of mice fed WD during a 4‐week intervention. We considered if adaptive responses to WD‐feeding are modified by concurrent exercise training and may be sex‐specific. We addressed these questions in male and female mice with 4 weeks of ad libitum access to a low‐fat or WD, or WD with concurrent treadmill training. We hypothesized that 4 weeks of WD would increase skeletal muscle mitochondrial respiratory capacity for both lipid and non‐lipid substrates compared with LFD, and that such effects would be greater with concurrent treadmill training.

## 2 METHODS

### 2.1 Animals and diets

**FIGURE 1:**
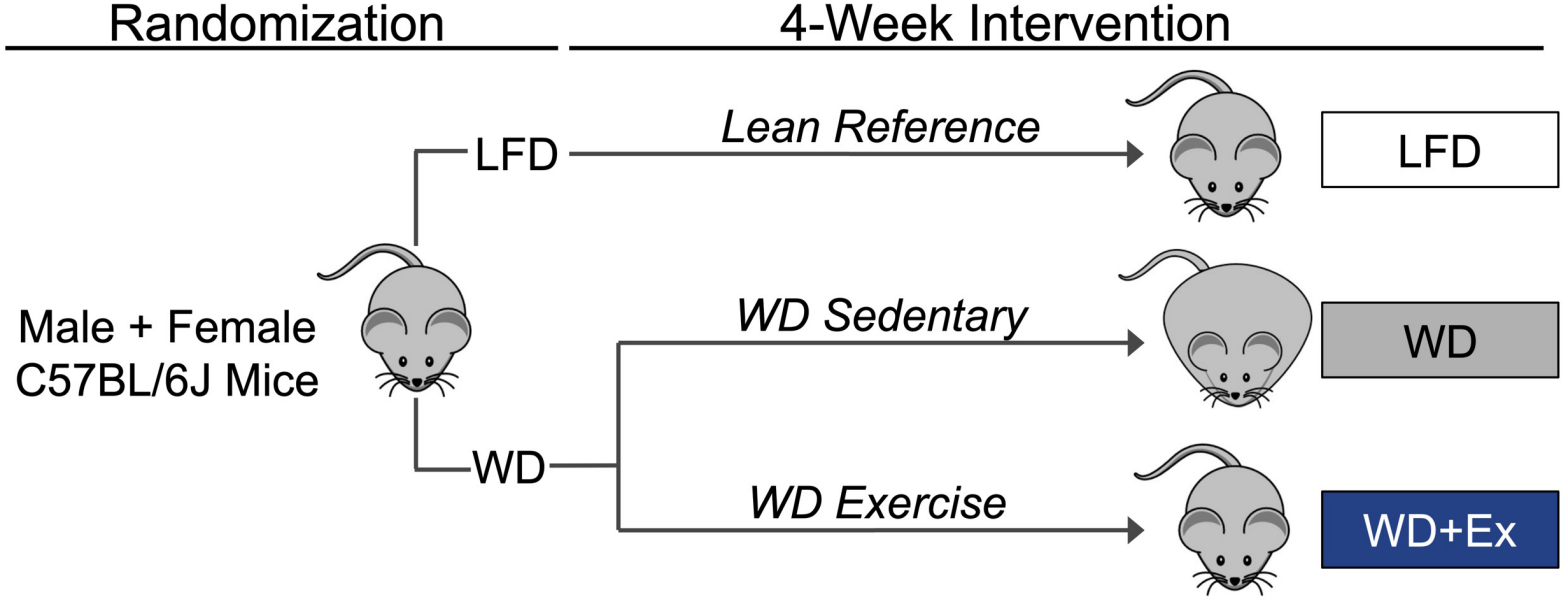
Study design. At 14 weeks of age, male and female C57BL/6J mice were randomized to receive ad libitum access to a western diet (WD) or low fat diet (LFD) for 4 weeks. WD mice (both male and female) were further randomized to perform graded aerobic exercise training (WD + Ex) for 4 weeks while the other groups remained sedentary. Mice in training groups were acclimated to the treadmill prior to the intervention. During week 3, mice were individually housed in metabolic cages for 48 h. During week 4, insulin tolerance tests and dual‐energy x‐ray absorptiometry were performed.

The study protocol was approved by the Animal Care and Use Committee at Oregon State University (#5164). The overall study design is depicted in Figure 1. Wild‐type C57BL/6J male and female mice were purchased from Jackson Laboratories (Bar Harbor, ME, USA) at 14 weeks of age. The mice were group‐housed four to five per cage in 12:12‐h light–dark cycle at 22°C with free access to food and water. Mice acclimated for at least 1 week before beginning the 4‐week study of diet and exercise. Mice consumed either a LFD (D14042701; Research Diets) or WD (D12079B; Research Diets). The percent kilocalories from total fat/carbohydrate/protein was 10/73/17 for the LFD (3.9 kcal/g) and 40/43/17 for the WD (4.7 kcal/g). Diet compositions are further detailed in Table 1. Mice within each diet group performed graded aerobic exercise training (Ex, described below) or remained sedentary. At the end of week 4, mice were euthanized 36 hours after the last bout of exercise and after a 4 h fast. Mice were anesthetized with isoflurane inhalation. Following loss of toe‐pinch and other reflexive behaviors, cardiac puncture was performed to collect plasma, with mice euthanized using cardiac excision. Tissue not used for high resolution respirometry was dissected then snap frozen in liquid nitrogen. Mice were 19 weeks of age at the end of the study. Group numbers for male mice were *n* = 4 (LFD), *n* = 7 (WD), *n* = 4 (WD + Ex), and *n* = 5 per group for female mice.

**TABLE 1:** Diet composition

Composition	LFD	WD
kcal (%)		
Protein	17	17
Carbohydrate	73	43
Fat	10	40
Ingredients (g/kg)		
Casein	195	195
DL‐Methionine	3	3
Corn Starch	695	50
Maltodextrin 10	150	100
Sucrose	0	341
Cellulose	50	50
Milk fat	42.5	200
Corn oil	10	10
Ethoxyquin	0.04	0.04
Mineral Mix	35	35
Calcium carbonate	4	4
Vitamin mix	10	10
Choline Bitartrate	2	2
Cholesterol	0	1.5
Total (kcal/g)	3.9	4.7

### 2.2 Exercise training

Exercise training was initiated concurrent with diet induction and progressed to 45 min per day on 5 days per week using a motorized treadmill (Panlab, Harvard Apparatus). Exercise training sessions were completed between 14:00 and 17:00 during the light cycle. During week 0, mice acclimated to the treadmill and exercise bouts began at 6 m/min at 0% incline for 5 min, then progressed to 9 m/min for 5 min and ended with 12 m/min for 5 min. In week 1, exercise bouts began at 12 m/min at 5% incline and progressed to 17 m/min for 45 min at 10% incline. During weeks 2, 3, and 4, mice exercised at 17 m/min for 45 min at 10% incline. Mice were encouraged to continue running with an air puff or mild shock plate at the back of the treadmill. Mice were removed from the treadmill when they refused to run, despite sitting on the shock grid for up to 5 seconds. Sedentary mice were not introduced to the treadmill but were routinely handled.

### 2.3 Blood glucose measurements and insulin tolerance testing

Blood glucose measurements and insulin tolerance tests (ITT) were performed in unrestrained mice at week 4. Mice were fasted and placed in individual cages with free access to water for 4 h prior to the ITT as previously described (Newsom et al., 2017). Body mass was recorded after the fasting period to calculate insulin dosing. Insulin (Humulin R; Eli Lilly) was provided with an intraperitoneal injection at 0.5 IU/kg body mass. Blood glucose was measured at 0, 15, 30, 45, 60, and 120 min. Blood glucose concentrations were measured by a handheld glucometer (Alphatrak2, Zoetis) from a tail vein nick and fall from baseline (FFB) was calculated in the first 30 min.

### 2.4 Whole body metabolic assessment

During Week 4, body composition was measured by dual‐energy X‐ray absorptiometry (Lunar PIXImus2, GE Healthcare) to quantify fat and lean mass. Mice were anesthetized using 2% isoflurane gas inhalation and sub‐cranial whole‐body images were acquired after 5 min. After 3 weeks of the intervention, metabolic rate was assessed by indirect calorimetry using a continuous metabolic monitoring system (Promethion, Sable Systems Int.) as previously described (Ehrlicher et al., 2020). Mice were singly housed in the metabolic cages immediately after an exercise bout and given ad libitum access to food and water for a 12‐h dark cycle to monitor whole‐body metabolism after exercise (or sedentary conditions). The mice remained in the cages with access to food for a full 48‐h light/dark cycle to measure whole‐body substrate metabolism in a rested condition. Air was sampled every 2 min from each cage to measure oxygen (O_2_) and carbon dioxide (CO_2_) content at a constant flow rate of 2000 ml/min. Energy expenditure and respiratory exchange ratio (RER) was calculated based on O_2_ consumption (VO_2_) and CO_2_ production (VCO_2_) as either 1‐ or 12‐h averages for each cage. Physical activity was continuously measured by a multidimensional beam break system and food intake and body mass were measured by electronic scales. Lean mass was used as a co‐variate for whole‐body metabolism as recommended (Tschöp et al., 2011).

### 2.5 Mitochondrial preparation and high resolution Respirometry

Mitochondria were isolated from freshly dissected quadriceps muscle complex (i.e., all 4 muscles) using differential centrifugation as previously described (Newsom et al., 2017). Muscle (~100 mg) was incubated in buffer A (100 mM KCl, 50 mM Tris base, 5 mM MgCl_2_‐6H_2_O, 1.8 mM ATP, and 1 mM EDTA, pH 7.2) containing protease (Subtilisin A, P5380, Sigma‐Aldrich) for 7 min on ice then homogenized for 10 min at 150 rpm in glass‐on‐glass homogenizers with 0.3 mm spacing between mortar and pestle. Samples were centrifuged for 5 min at 750 × *g* and 4°C to collect the myofibrillar proteins. The supernatant was centrifuged for 5 min at 10,000 × *g* and 4°C to pellet the mitochondria. The supernatant was discarded and the mitochondrial pellet was washed with buffer A and centrifuged 5 min at 9000 × *g* and 4°C. The supernatant was discarded and the mitochondria were resuspended in 1:4.2 (w/v) buffer B (180 mM sucrose, 35 mM KH_2_PO_4_, 10 mM Mg‐Acetate, 5 mM EDTA, pH = 7.5 at room temperature).

High‐resolution respirometry was performed using two Oxygraph‐2 k units (Oroboros Instruments) with MiR05 respiration buffer (0.5 mM EGTA, 3 mM MgCl_2_‐6‐H_2_O, 60 mM lactobionic acid, 20 mM taurine, 10 mM KH_2_PO_4_, 20 mM HEPES, 110 mM sucrose, and 1 g/L bovine serum albumin, BSA) at 37°C while stirring at 750 rpm. Protocols were performed to measure oxygen consumption and concurrent hydrogen peroxide (H_2_O_2_) emission (10 μM amplex red, 5 U/mL superoxide dismutase and 1 U/mL horseradish peroxidase). We measured H_2_O_2_ during O_2_ as previously reported (Ehrlicher et al., 2020; Newsom et al., 2017; Robinson et al., 2019). The H_2_O_2_ detection chemicals were added to the chambers containing MIR05 respiration medium at the beginning of the experiment, prior to the addition of the mitochondrial suspension, similar to as previously described (Robinson et al., 2019). Each protocol was performed in duplicate chambers on one machine and the rate of oxygen consumed (JO_2_, pmol/(s∙ml)) was calculated as the average of values from the two 2.0 ml chambers. O_2_ flux and H_2_O_2_ emission was determined from steady‐state flux and adjusted for instrumental background. H_2_O_2_ flux decreases at lower oxygen concentrations and our measured H_2_O_2_ may be influenced by changing O_2_ concentrations in the chambers (which ranged from 30 to 190, with most measures between 60 and 100 μM) (Stepanova et al., 2019). Respirometers had routine calibration procedures to determine instrumental O_2_ background by measuring steady state JO_2_ at 4 steps of O_2_ concentrations from ambient to zero using manufacturer recommendations. These corrections were applied to every run to correct oxygen flux rates for instrument components (Doerrier et al., 2018; Dykens & Will, 2008). Each run included air calibration at ambient O_2_ concentration (e.g. open chamber) at 37°C then a stirrer test to verify O_2_ sensor had monophasic response to changing O_2_ concentrations when stir bars were stopped and restarted. We used H_2_O_2_ injections throughout each run (prior to injection of mitochondria and after succinate) to account for changes in sensitivity of Amplex Red reaction over time (Makrecka‐Kuka et al., 2015). Oxygen consumption is presented as a measure of mitochondrial quantity and capacity (pmol/(s∙ml)). We used Pierce BCA assay (Thermo Fisher Scientific) to determine protein concentration of the mitochondrial preparation in each suspension (average ~ 1.9 μg/μl). Normalized respiration to protein abundance in the respiratory chamber was used to determine intrinsic mitochondrial function (i.e., mitochondrial quality) (pmol/(s∙μg)), see Data S1.

The titration protocol measured oxidative phosphorylation (OXPHOS), oligomycin‐induced dissipation (LEAK), and noncoupled respiration. Addition of the mitochondrial suspension (50 μl) was followed by ADP (5 mM). Substrate additions were characterized by their ability to donate electrons to the electron transfer system (ET), including NADH (N‐linked) via complex I, succinate (S‐linked) via complex II, and fatty acid (F‐linked) via electron transferring flavoprotein complex and complex I. We conducted two independent protocols using lipid substrate (octanoyl‐carnitine and malate [OCM]), followed by non‐lipid substrates (glutamate and succinate), or only non‐lipid substrates (glutamate, malate and succinate, [GMS]) to determine rates of oxygen consumption (JO_2_). Octanoylcarnitine was prepared in accordance with manufacturer recommendations (Oroboros Instruments) to achieve a kinetically saturating concentration of 0.5 mM, which aligns with previous protocols of fatty acid titrations below 60 μM (Petrick & Holloway, 2019). To verify our titration injection saturated respiration, we performed titration protocols of sequential 10 μM increases in chamber concentrations (up to 50 μM) then injection to raise chamber concentration of 0.5 mM (data not shown). These experiments reveal little increase in respiration beyond 50 μM and indicate 0.5 mM is a kinetically saturating dose for octanolycarnitine in isolated mitochondria. An underlying purpose of completing both protocols was to determine the impact of lipid‐supported respiration on respiration of non‐lipid substrates, as the only difference between the OCM and GMS protocols was the presence of octanoyl‐carnitine. We measured LEAK respiration following maximal stimulation of oxidative phosphorylation with the addition of the ATP synthase inhibitor, oligomycin. We then measured non‐coupled electron transfer respiration via the addition of the protonophore, carbonyl cyanide *p*‐(trifluoromethoxy) phenylhydrazone. The complex III inhibitor, Antimycin A, was added to measure non‐mitochondrial respiration. Primary chemical reagents were purchased from Millipore Sigma. Detailed protocols are provided in Table 2.

**TABLE 2:** High‐resolution respirometry protocols

Addition	Concentration	Description
Octanoyl‐Carnitine + Malate (OCM)		
Amplex red	10 μM	H_2_O_2_ emission reagent
Horseradish peroxidase	1 U/ml	H_2_O_2_ emission reagent
Superoxide dismutase	5 U/ml	H_2_O_2_ emission reagent
Isolated mitochondria^a^		
Octanoly‐carnitine	0.5 mM	F‐linked substrate
Malate	2 mM	N‐linked substrate
ADP	5 mM	ATP‐synthase substrate
Glutamate	10 mM	N‐linked substrate
Succinate	10 mM	S‐linked substrate
Cytochrome C	10 μM	Mitochondrial membrane integrity
Oligomycin	2.5 μM	ATP‐synthase inhibitor
FCCP^b^	0.5 μM^c^	Protonophore
Antimycin A	2.5 μM	Complex III inhibitor
Glutamate + Malate + Succinate (GMS)		
Amplex red	10 μM	H_2_O_2_ emission reagent
Horseradish peroxidase	1 U/ml	H_2_O_2_ emission reagent
Superoxide dismutase	5 U/ml	H_2_O_2_ emission reagent
Isolated mitochondria^a^		
Glutamate	10 mM	N‐linked substrate
Malate	2 mM	N‐linked substrate
ADP	5 mM	ATP‐synthase substrate
Succinate	10 mM	S‐linked substrate
Cytochrome C	10 μM	Mitochondrial membrane integrity
Oligomycin	2.5 μM	ATP‐synthase inhibitor
FCCP^b^	0.5 μM^c^	Protonophore
Rotenone	0.5 μM	Complex I inhibitor
Antimycin A	2.5 μM	Complex III inhibitor

^a^ Mitochondria were isolated from quadriceps muscle via centrifugation and resuspended in buffer B.

^b^ Carbonyl cyanide *p*‐(trifluoromethoxy) phenylhydrazone.

^c^ Sequential 0.5 μM additions to achieve maximal noncoupled respiration (Robinson et al., 2019).

### 2.6 Immunoblotting

Quadriceps muscle (~30 mg) was homogenized, incubated at 4°C for 20 min, and centrifuged at 10,000 × *g* for 10 min at 4°C in preparation for immunoblotting as described (Newsom et al., 2017). Approximately 30 μg of whole‐cell lysate protein was resolved on 8%–12% Bis‐Tris gels and transferred to nitrocellulose membranes. Control samples were loaded at the beginning and end of each gel to serve as an internal control, with the average intensity of control bands used to normalize band intensities among gels. Ponceau staining of membranes and detection of α‐tubulin (#926–42213, Licor) was performed to verify equal loading and transfer of protein to the nitrocellulose membrane. Membranes were blocked in 5% BSA in Tris‐buffered saline +1% Tween (TBST). Primary chemical reagents were purchased from Millipore Sigma Aldrich (St. Louis) and Bio‐Rad. Primary antibodies were diluted in 5% BSA‐TBST and membranes incubated overnight at 4°C. Primary antibodies included oxidative phosphorylation (OXPHOS) cocktail (#110413), hydroxyacyl‐CoA dehydrogenase (HADH, #154088), carnitine palmitoyltransferase I (CPT1, #134988), and mitochondrial transcription factor A (TFAM, #47517) that were purchased from Abcam. Peroxisome proliferator‐activated receptor‐gamma coactivator 1‐alpha (PGC1α, #PA572948) was purchased from Invitrogen. Fatty acid translocase (CD36, #50559214) was purchased from Proteintech. Secondary antibodies (IRDye 800CW and 680 IgG, Licor) were diluted in 5% BSA‐TBST. Membranes incubated at room temperature for 1 h. Images were generated using infrared detection (Odyssey, Licor) and analyzed using Image Studio software (Licor). Images were collected using a moderate‐intensity laser power without indication of oversaturation, indicating densitometry was performed within the wide linear range of the instrument.

### 2.7 Statistical analysis

Comparisons among groups within each sex were performed using a repeated measures two‐way (group × time) analysis of variance (ANOVA) or one‐way (group) ANOVA, with Sidak's multiple comparisons test between LFD and WD (diet effect) and between WD and WD + Ex (exercise effect), when appropriate. Main effects of time are not reported. Energy expenditure data were analyzed using analysis of covariance (ANCOVA) of lean body mass and treatment as described previously (Newsom et al., 2017; Tschöp et al., 2011). Statistical analysis was performed using Prism version 8 (GraphPad Software) and JMP Pro version 15.0 (SAS Institute). Statistical significance was set as *p* < 0.05. Figures were generated using Prism version 8 (GraphPad Software). Data are expressed as mean ± SEM. Based on our previous reports of HFD and LFD feeding in male C57BL/6J mice, lipid respiration normalized for mitochondrial protein content for LFD mice was mean (SD) of 98.25 (22.9) and HFD was 189 (66.92) JO_2_ (pmol/(s∙μg)) (Ehrlicher et al., 2020). Using *n* = 5 per group, assuming a SD of 44, α at 0.05 and 1‐β of 0.5, an unpaired t‐test could detect differences of 62 JO_2_ (pmol/(s∙μg)).

## 3 RESULTS

### 3.1 Body mass and composition

**FIGURE 2:**
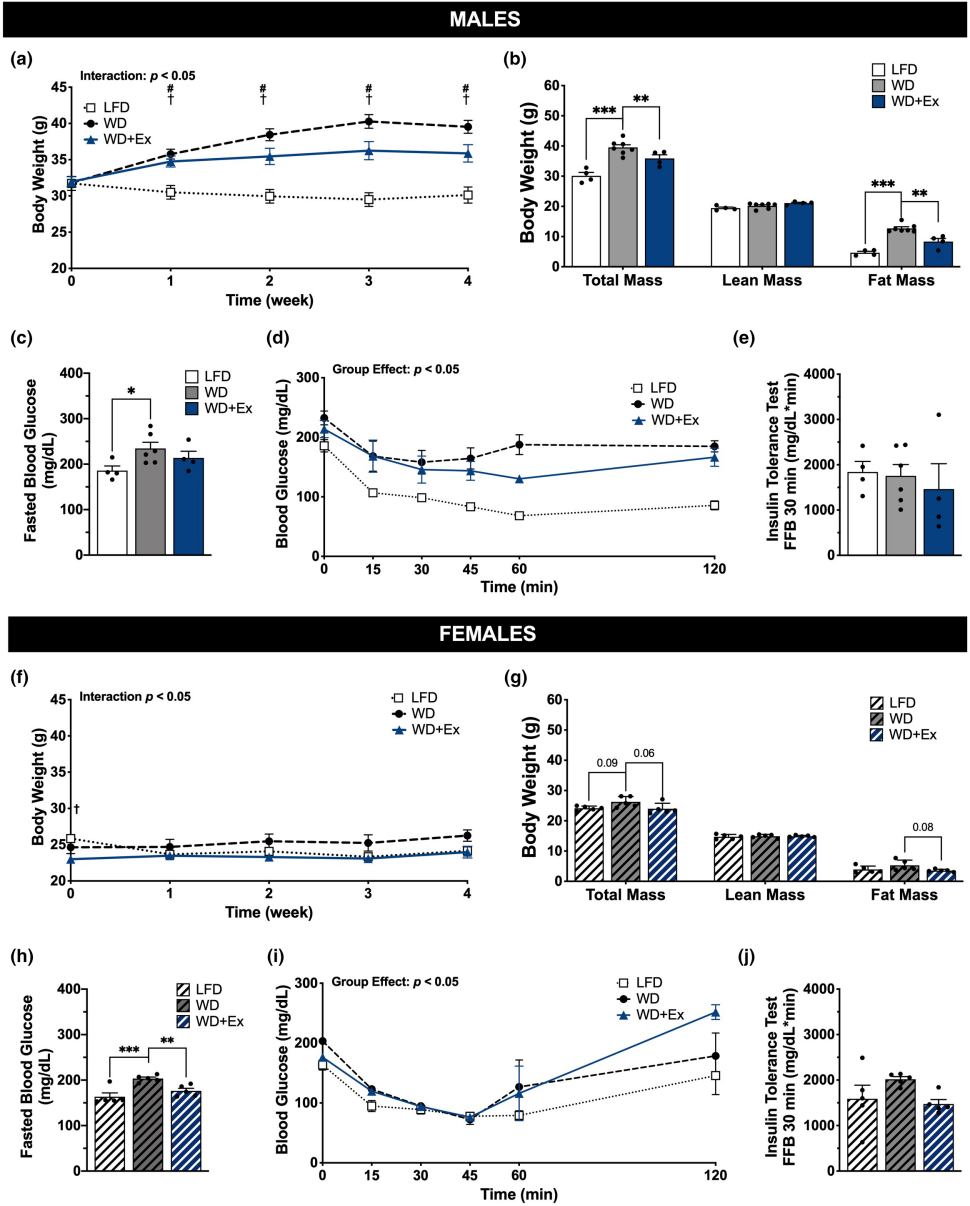
Mouse characteristics. (a/f) Male and female body mass (g) measured weekly over 4 weeks. (b/g) Total mass with lean and fat mass (grams) measured during week 4 of the intervention. (c/h) 4 h fasted blood glucose at week 4. (d/i) Blood glucose detected by tail vein blood during insulin tolerance test after 4 h fasting at baseline, 15, 30, 45, 60, and 120 min after intraperitoneal injection of insulin with quantification of fall from baseline (FFB) during week 4 (e/j). Repeated measure ANOVA was used to test for interaction and group effects. # denotes *p* < 0.05 WD compared with LFD. † denotes *p* < 0.05 WD + Ex compared with LFD. One‐way ANOVA was used for statistical analysis with Sidak's multiple comparisons test when appropriate. *, **, and *** denote *p* < 0.05, *p* < 0.01, and *p* < 0.001, respectively. Data are presented as mean ± SEM, *n* = 4–7 per group.

Male mice had progressive mass gain during 4‐weeks of WD feeding that resulted in greater total body mass compared with LFD mice (*p* < 0.05, Figure 2a,b), which was due to greater fat mass (*p* < 0.05, Figure 2b). Concurrent exercise training during WD (WD + Ex) attenuated but did not prevent mass gain (Figure 2a), due to lower fat mass compared with WD mice (*p* < 0.001, Figure 2b). Save for modest yet statistically significant differences in baseline body mass, female mice had lesser, non‐significant changes in body mass and composition as a function of the interventions (Figure 2f,g). As a result of the progressive aerobic training protocol, male mice ran 2536 ± 182 m and female mice ran 2889 ± 302 m in week 4 (full training dataset provided in Figure S4).

### 3.2 Whole‐body metabolic characteristics

Males fed WD had higher fasting blood glucose concentrations compared with LFD (*p* < 0.05, Figure 2c). Female mice fed WD also had higher fasting blood glucose concentrations than mice fed LFD, whereas WD + Ex had lower fasting blood glucose concentrations compared with WD (both comparisons *p* < 0.05, Figure 2h). Such differences in fasting glucose contributed to a main effect of group for both male and female mice during the ITTs (Figure 2d,i). However, there was no significant difference in insulin tolerance calculated during the first 30 min for either male or female mice (Figure 2e,j, respectively).

**FIGURE 3:**
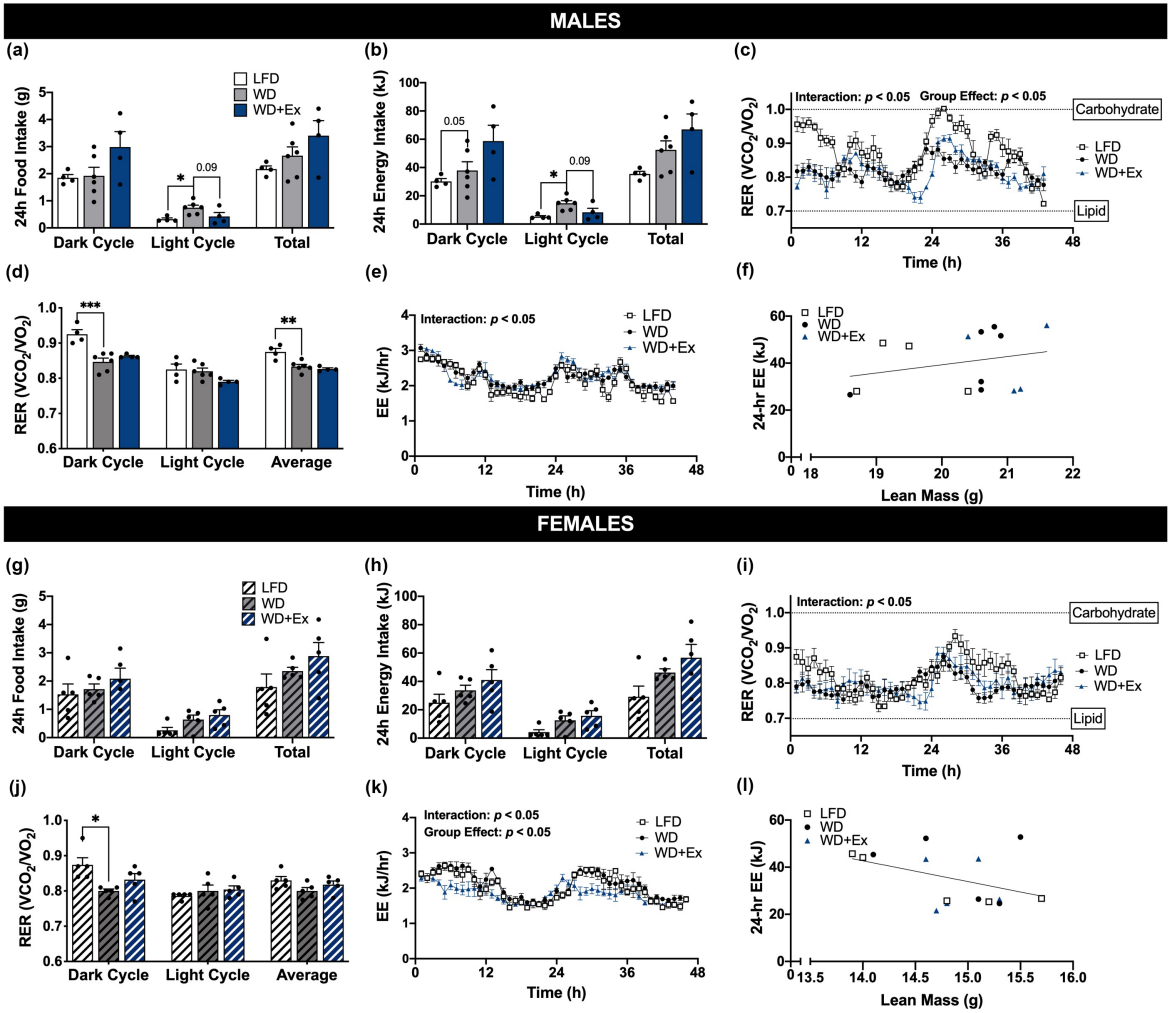
Whole body metabolic phenotype. Mice were individually housed in metabolic cages for 48 h to assess food intake, substrate oxidation and energy expenditure during week 3. Food intake is presented in grams (a/g) and energy intake in kilojoules (b/h) during the second 24‐h time period. indirect calorimetry was used to calculate respiratory exchange ratio (RER) (c/i) 1‐h averages and d/j) 12‐h averages as well as energy expenditure (EE) (e/k) 1‐h averages and (f/l) 24‐h averages. Repeated measure ANOVA was used to test for interaction and group effects. One‐way ANOVA and ANCOVA was used for statistical analysis with Sidak's multiple comparisons test when appropriate. *, **, and *** denote *p* < 0.05, *p* < 0.01, and *p* < 0.001, respectively. Data are presented as mean ± SEM, *n* = 4–7 per group.

Mice were individually housed in metabolic cages to examine energy intake and whole‐body energy expenditure. Males fed a WD had increased energy intake during the light cycle compared with LFD (*p* < 0.05, Figure 3b). As expected, RER was lower (indicating increased reliance on fat oxidation) among mice fed the WD compared with LFD. Such effects were most pronounced during the dark cycle (i.e., primary feeding time) for both males and females (Figures 3c/d and i/j, respectively). There was a group × time interaction for energy expenditure (kJ/h) among both male and female mice (both *p* < 0.05, Figures 3e/k). However, there was no significant effect on the relationship between lean body mass and energy expenditure when measured during 24 h of feeding among male or female mice (Figure 3f,l, respectively).

### 3.3 Skeletal muscle mitochondrial respiration

**FIGURE 4:**
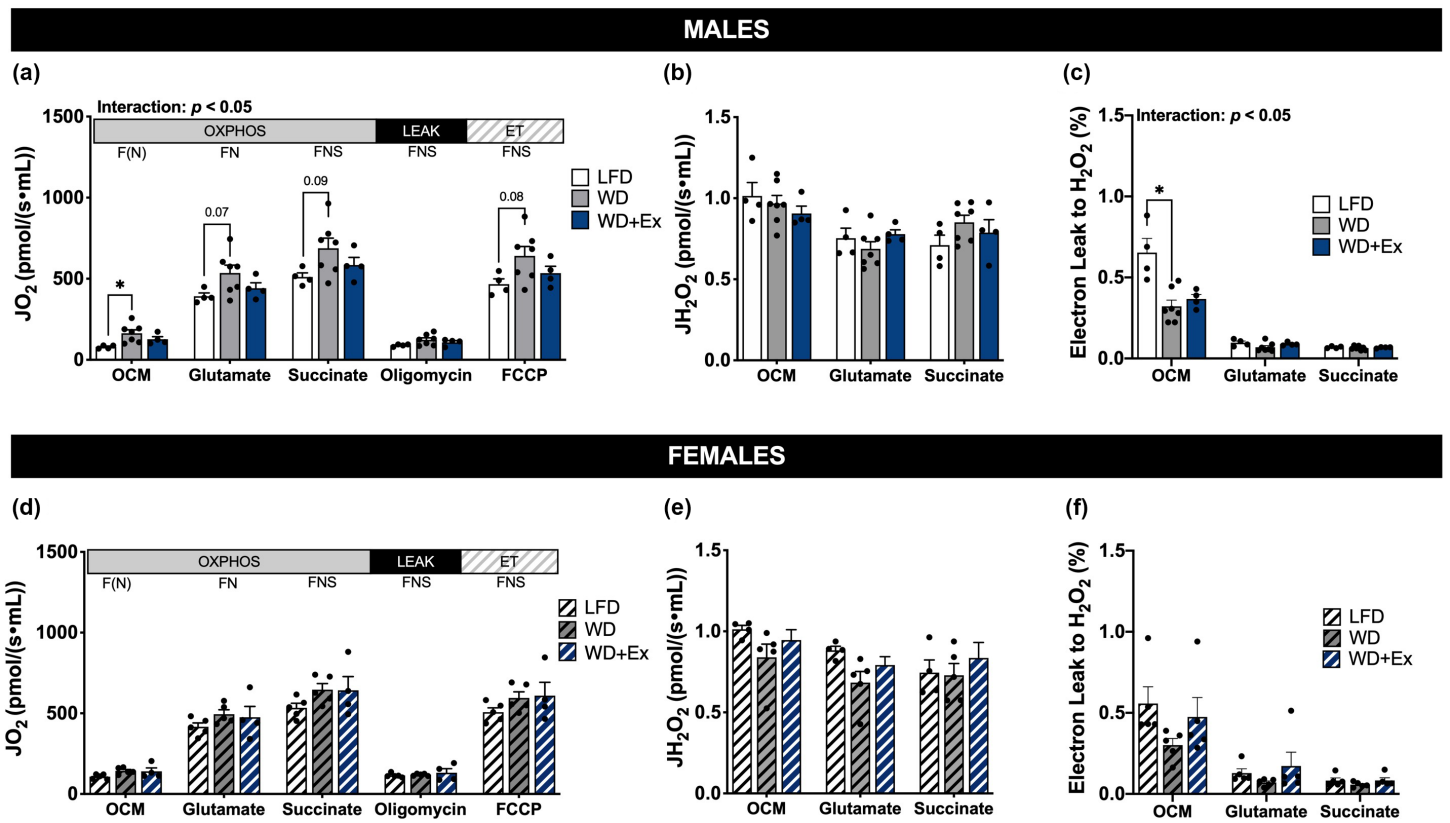
Combined lipid and non‐lipid supported skeletal muscle mitochondrial respiration. Quadricep muscles were collected after 4‐h fast. (a/d) Respiration of isolated mitochondria with non‐limiting ADP. JO_2_ is rate of oxygen consumption (pmol/(s∙ml)). Octanoyl‐carnitine (OC) is F‐linked (F), donating electrons to electron‐transferring flavoprotein complex and complex I; malate (M) and glutamate are N‐linked (N), donating electrons to complex I; succinate is S‐linked (S), donating electrons to complex II. Substrate‐linked oxidative phosphorylation (OXPHOS), oligomycin‐induced leak (LEAK), and non‐coupled electron transfer (ET) is shown. (b/e) Rates of H_2_O_2_ emission with non‐limiting ADP expressed relative to tissue mass (pmol H_2_O_2_/(s∙ml)). (c/f) Electron leak to H_2_O_2_ calculated from simultaneous measurement of H_2_O_2_ emission and JO_2_ during oxidative phosphorylation. FCCP, carbonyl cyanide p‐(trifluoromethoxy) phenylhydrazone. Repeated measure ANOVA with Sidak's multiple comparisons test was used to test for interaction and group effects. *p < 0.05. Data are presented as mean ± SEM, *n* = 4–7 per group.

**FIGURE 5:**
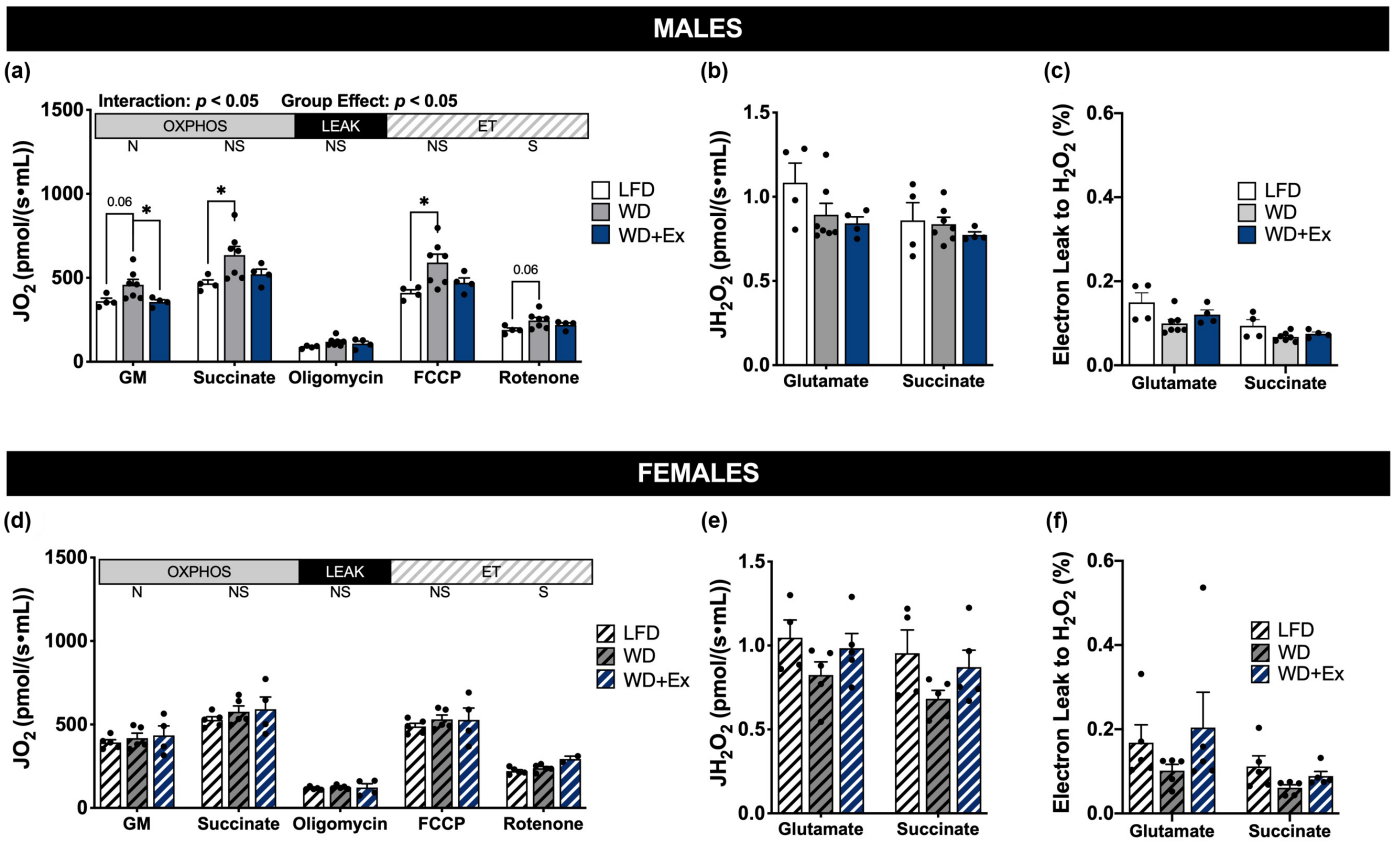
Non‐lipid supported skeletal muscle mitochondrial respiration. Quadricep muscles were collected after 4‐h fast. (a/d) Respiration of isolated mitochondria with non‐limiting ADP. JO_2_ is rate of oxygen consumption (pmol/(s∙ml)). Malate (M) and glutamate (G) are N‐linked (N), donating electrons to complex I; succinate is S‐linked (S), donating electrons to complex II. Substrate‐linked oxidative phosphorylation (OXPHOS), oligomycin‐induced leak (LEAK), and non‐coupled electron transfer (ET) is shown. (b/e) Rates of H_2_O_2_ emission with non‐limiting ADP expressed relative to tissue mass (pmol H_2_O_2_/(s∙ml)). (c/f) Electron leak to H_2_O_2_ calculated from simultaneous measurement of H_2_O_2_ emission and JO_2_ during oxidative phosphorylation. FCCP, carbonyl cyanide p‐(trifluoromethoxy) phenylhydrazone. Repeated measure ANOVA with Sidak's multiple comparisons test was used to test for interaction and group effects. *p < 0.05. Data are presented as mean ± SEM, *n* = 4–7 per group.

We determined mitochondrial respiration to examine differences in skeletal muscle respiratory capacity. A significant group × time interaction among male mice revealed WD mice had greater fatty acid supported respiration compared with LFD mice (*p* < 0.05 for OCM, Figure 4a). Male WD mice also tended to have greater rates of oxidative phosphorylation compared with LFD during subsequent additions of glutamate and succinate (*p* = 0.07 and *p* = 0.09, respectively, Figure 4a), as well as a tendency for greater rates of noncoupled electron transfer compared with LFD (*p* = 0.08, Figure 4a). A significant group × time interaction and main effect of group were also present during respiration of non‐lipid substrates (Figure 5a), suggesting increases in respiratory capacity among WD mice compared with LFD for both lipid and non‐lipid substrates. Concurrent exercise training in male WD‐fed mice had limited impact on measures of skeletal muscle respiratory function, with perhaps a modest effect to limit the increase in respiratory capacity compared with WD alone. Normalizing respiration rates to mitochondrial protein content (to examine mitochondrial quality) attenuated group‐based differences in respiration (Figures S1a and S2a); however, lipid‐supported respiration remained elevated in WD compared with LFD mice (*p* = 0.09 for OCM, Figure S1a).

In contrast to the observations among male mice, there were no differences in respiration among female mice during either respiration protocol, presented as a function of tissue mass or relative to protein content (Figures 4d and 5d; Figures S1c and S2c). The addition of cytochrome *c* did not significantly alter respiration in any protocol (i.e., changes in JO_2_ < 10%), indicating that mitochondrial outer membranes were largely intact (data not shown).

### 3.4 Skeletal muscle mitochondrial H_2_O_2_ emission

Reactive oxygen species are implicated in oxidative stress with high dietary fat, we therefore performed simultaneous measurement of H_2_O_2_ emission during respiratory protocols. There were no significant differences in H_2_O_2_ emission among male or female groups during states of oxidative phosphorylation using lipid and non‐lipid substrates (Figures 4b/e and 5b/e). Electron leak to H_2_O_2_ was lower among male WD mice compared with LFD during lipid‐supported respiration (*p* < 0.05 for OCM, Figure 4c), driven by the increase in respiration (Figure 4a). However, H_2_O_2_ emission relative to protein content was lower among male and female mice fed the WD compared with LFD during both respiration of both lipid and non‐lipid substrates (Figures S1b/d and S2b/d). H_2_O_2_ emission measurements were not made during LEAK or noncoupled electron transfer due to the addition of cytochrome *c*.

### 3.5 Skeletal muscle mitochondrial protein content

**FIGURE 6:**
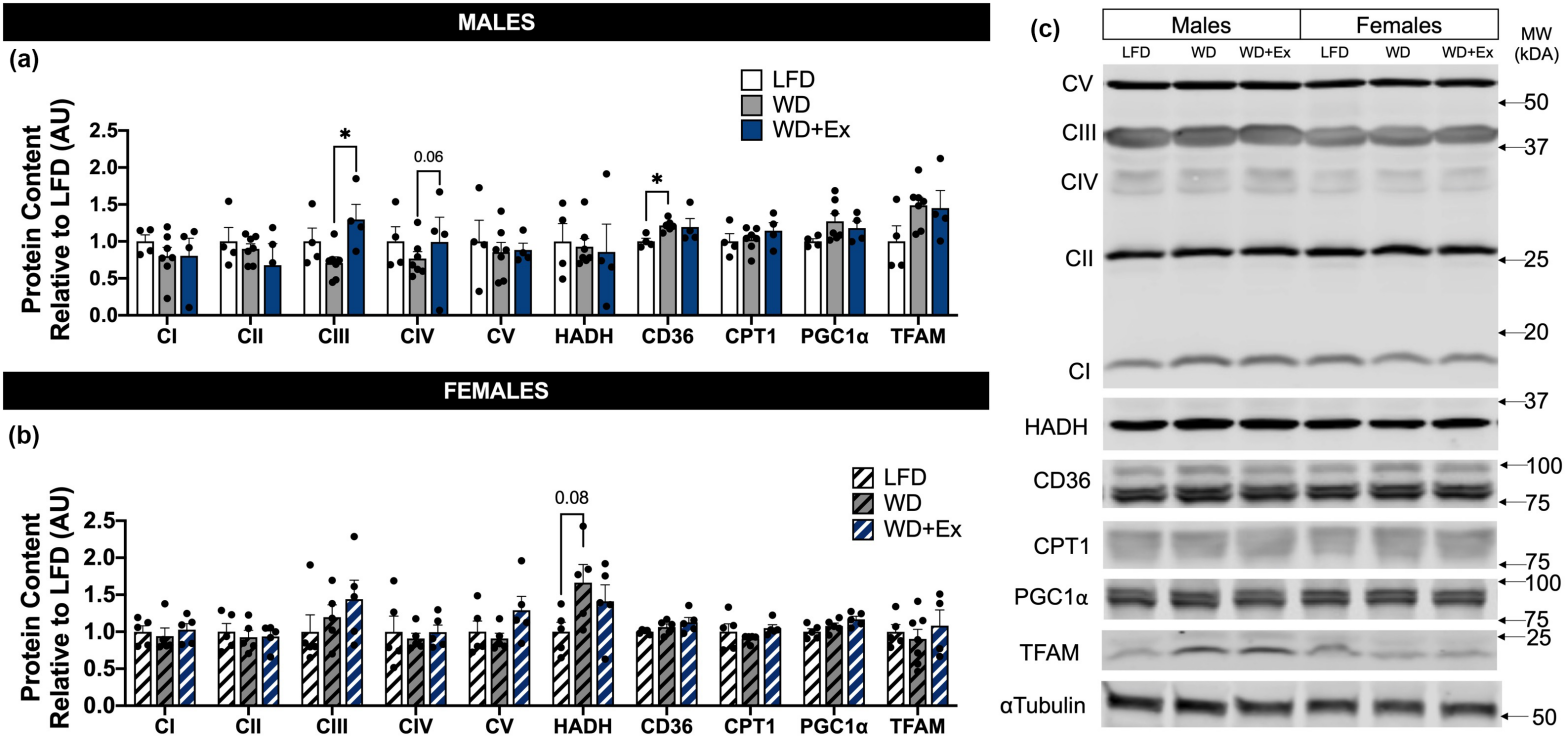
Mitochondrial protein content after WD and exercise training. (a/b) Western blots were used to estimate the content of mitochondrial proteins as well as proteins involved in mitochondrial biogenesis and mitochondrial lipid metabolism. The complex of subunits of the electron transfer system, CI, CII, CIII, CIV; and ATP Synthase, CV. Hydroxyacyl‐CoA dehydrogenase, HADH; fatty acid translocase, CD36; carnitine palmitoyltransferase 1, CPT1; peroxisome proliferator‐activated receptor‐gamma coactivator 1⍺, PGC1⍺; mitochondrial transcription factor A, TFAM. Protein content expressed as arbitrary units relative to LFD. (c) Representative Western blot images including ⍺Tubulin. One‐way ANOVA was used for statistical analysis with Sidak's multiple comparisons test when appropriate. *p < 0.05. Data are presented as mean ± SEM, *n* = 3–7 per group.

We determined if diet and exercise interventions influenced skeletal muscle mitochondrial protein content, focusing on mitochondrial complex content, regulators of mitochondrial biogenesis and lipid oxidation. Male WD + Ex mice had significantly greater complex III subunit content compared with WD (*p* < 0.05, Figure 6a). No significant differences in mitochondrial complex content were noted among female mice (Figure 6b). Male WD mice had greater skeletal muscle content of fatty acid transporter CD36 compared with LFD mice (*p* < 0.05, Figure 6a). There was a tendency for higher HADH content in female WD mice compared with LFD mice (*p <* 0.05, Figure 6b). There were no statistically significant differences in CI, CII, CIV, ATP synthase, CPT1, or TFAM among male or female mice.

## 4 DISCUSSION

We investigated sex‐specific changes in whole‐body metabolism and skeletal muscle mitochondrial respiration during 4 weeks of WD feeding, with or without concurrent exercise training. We hypothesized that 4 weeks of WD feeding would result in greater skeletal muscle mitochondrial respiratory function for both lipid and non‐lipid substrates compared with LFD. We determined skeletal muscle mitochondrial capacity for lipid and non‐lipid substrates was greater after short‐term WD compared with LFD, but only among male mice. Short‐term concurrent treadmill exercise training during WD had limited impact on skeletal muscle respiratory function respiration in male or female mice. The primary findings indicate that 4 weeks of WD feeding can stimulate greater skeletal muscle mitochondrial oxidative capacity of both lipid and non‐lipid substrates in male but not female mice.

Four weeks of WD feeding led to greater skeletal muscle respiratory capacity for both lipid and non‐lipid substrates among male mice (Figures 4 and 5). Normalizing for mitochondrial protein content eliminated the increase in respiration of non‐lipid substrates, whereas the capacity for respiration of lipid remained elevated compared with LFD (Figures S1 and S2). We interpret these results to indicate WD increased overall skeletal muscle respiratory capacity, with adaptations to increase intrinsic capacity for lipid respiration. In agreement with our findings, diets with 50% kcal from fat have been shown to induce notable increases in the capacity for fat oxidation in skeletal muscle within 4 weeks (Hancock et al., 2008). A key finding from Hancock et al., was that male Wistar rats fed the HFD had numerous measures to indicate increased mitochondrial content compared with skeletal muscle from rats fed a LFD, with significant remodeling occurring after 3–4 weeks of diet intervention. Because increased rates of palmitate oxidation (in whole‐cell homogenates) among the HFD rats were normalized to tissue mass, it is likely that the greater capacity for palmitate oxidation was driven, at least in part, by the increase in mitochondrial density. We observed limited changes in mitochondrial proteins in the current study, yet our respiration data suggest mitochondrial content may be increasing with 4‐weeks of WD compared with LFD.

Female mice exhibited almost no changes in skeletal muscle mitochondrial respiratory function in this study. An interesting consideration is that diet‐induced changes in skeletal muscle respiratory capacity may be linked with excessive energy intake. For example, female mice consuming the WD were not in a state of significant positive energy balance as indicated by minimal change in body mass and composition (Figure 2f/g). Such findings are in agreement with previous reports of protection against diet‐induced obesity in female mice (Hwang et al., 2010; Ingvorsen et al., 2017; Pettersson et al., 2012). Conversely, male WD mice exhibited mass gain (i.e., positive energy balance) and greater skeletal muscle respiratory capacity compared with LFD, whereas male WD + Ex mice had attenuated mass gain concurrent with tempered changes in skeletal muscle respiratory function. Furthermore, genetically hyperphagic rats fed a low‐fat chow diet have demonstrated an increased mitochondrial oxidative capacity (Holloway et al., 2009). The current experiment was not designed to test the causality of changes in energy balance as the driver of adaptive responses to skeletal muscle mitochondrial function. Controlling caloric intake will help separate the effects of different diet compositions from positive energy balance on regulation of skeletal muscle respiratory function. These are important future directions that may help to explain differential responses among male and female mice observed here.

Another important consideration is known differences in skeletal muscle respiratory function and overall fat metabolism between males and females, including increased mitochondrial oxidative capacity among females (Cardinale et al., 2018; Miotto et al., 2018; Montero et al., 2018). In agreement with previous reports, female mice in the current study exhibited higher capacity for lipid‐supported mitochondrial respiration compared with males during LFD feeding (107.6 ± 6.5 vs 79.3 ± 5.3 JO_2_ pmol/(s∙ml), respectively; Figure 4). Further to this point, RER appeared to be lower among female compared with male mice regardless of group, indicating increased reliance upon fat oxidation compared with males. It is therefore possible the increase in fat content of the WD compared with LFD was not sufficient to cause significant remodeling of skeletal muscle mitochondria among female mice.

An unexpected result was the lack of change in skeletal muscle respiratory function with aerobic exercise training among both male and female mice. Running distance was not matched between sexes so we cannot directly compare response to absolute workload between sexes, particularly since males were heavier which increases work when running at an incline. We have previously shown that 8 weeks of treadmill exercise training during HFD increased skeletal muscle mitochondrial respiratory capacity for both lipid and non‐lipid substrates, even after normalizing to mitochondrial protein content (Ehrlicher et al., 2020). In the present study, we found that 4 weeks of treadmill exercise training (with concurrent WD) generally failed to increase mitochondrial respiratory capacity compared with WD alone (Figures 4 and 5). This suggests the training duration or intensity were not sufficient to induce significant changes in mitochondrial function. Short term aerobic exercise training (4–5 weeks) in mice has been shown to induce mitochondrial turnover (Chen et al., 2018; Lira et al., 2013). The training intervention used in these studies was voluntary wheel running during which mice run further (>8 km/day) than forced treadmill training. The lower training stimulus of treadmill training and possibility of stress of treadmill (noise, disturbed circadian rhythm, changing environment) versus voluntary wheel running likely contributes to attenuated skeletal muscle mitochondrial adaptations. Respiration normalized to mitochondrial protein content was similar among groups in the present study (Figures S1 and S2), indicating preservation of mitochondrial quality. An important future consideration is to use a more intact system, such as permeabilized muscle fibers, to further determine the effect of WD on mitochondrial quantity and function in an environment modulated by intracellular interactions.

We report H_2_O_2_ during steady state JO_2_ with O_2_ concentrations approximately 60–100 μM. Previous studies demonstrate O_2_ concentrations are important to consider when measure H_2_O_2_ during respirometry (Li Puma et al., 2020) and we acknowledge that JH_2_O_2_ would be slightly higher if O_2_ was maintained at steady concentrations. Both male and female mice had lower rates of mitochondrial H_2_O_2_ emission following the WD compared with LFD when normalized to mitochondrial protein (Figures S1 and S2). Our previous investigations using longer‐duration HFD indicated lower electron leak to H_2_O_2_ compared with LFD among male mice (Ehrlicher et al., 2020). The current findings in male mice replicate this finding (Figure 4c), and were similarly driven by increased oxidative capacity and unchanged rates of H_2_O_2_ emission relative to tissue mass. The implication of these findings is that short‐term WD feeding led may improve mitochondrial regulation of electron flow when compared with LFD feeding (i.e., tighter coupling with reduction of oxygen to water vs. generation of reactive oxygen species). An important future direction will be to determine the impact of short‐term WD on regulation of mitochondrial quality control processes (e.g., fission, fusion, mitophagy) that may underlie the observed changes in electron flow.

In summary, 4 weeks of WD induced mass gain and modest physiologic adaptation of skeletal muscle mitochondrial respiratory function among male mice, whereas female mice were protected from mass gain and resistant to changes in mitochondrial respiratory capacity. Short‐term concurrent aerobic exercise training did not result in inducing greater mitochondrial respiratory capacity in WD‐fed mice. Important future directions will be to determine to what extent diet‐induced changes in skeletal muscle mitochondrial respiration are linked with dietary composition compared with energetic availability, including how differences in energy balance contribute to sex‐based differences in regulation of skeletal muscle mitochondrial remodeling.

## AUTHOR CONTRIBUTIONS

E.M.M performed analysis, figure development, and drafted the manuscript. S.E.E performed animal procedures and data collection. H.D.S., M.M.R. and S.A.N. assisted with planning the study, performing animal procedures and data collection. All authors contributed to data interpretation, manuscript editing and approved the final version of the manuscript.

## FUNDING INFORMATION

The current project was supported by funds provided to M.M.R. and S.A.N. by Oregon State University. Additionally, M.M.R. was supported by K01DK103829 from the National Institutes of Health, S.A.N. was supported by KL2TR002370 as part of the Oregon Clinical & Translational Research Institute Clinical Translational Science Award and UL1TR002371 from the National Institutes of Health. E.M.M., S.E.E. and H.D.S. were supported by assistantships provided by Oregon State University.

## CONFLICT OF INTEREST

The authors declare that the research was conducted in the absence of any commercial or financial relationships that could be construed as a potential conflict of interest.

## Supporting information


Figures S1–S4
