# Supplementary material for: Impact of 4 weeks of western diet and aerobic exercise training on whole‐body phenotype and skeletal muscle mitochondrial respiration in male and female mice
Source: Physiol Rep. 2022 Dec 21;10(24):e15543. doi: 10.14814/phy2.15543 (PMC9768729; doi:10.14814/phy2.15543)

Supplementary Material

Supplementary Figure 1. Lipid supported skeletal muscle mitochondrial respiration. Quadricep muscles were collected after 4-h fast. **A/D)** Respiration of isolated mitochondria with non-limiting ADP. Rates of oxygen consumption (JO_2) are expressed relative to protein content (pmol/(s·µg)). Octanoyl-carnitine (OC) is F-linked (F), donating electrons to electron-transferring flavoprotein complex and complex I; malate (M) and glutamate are N-linked (N), donating electrons to complex I; succinate is S-linked (S), donating electrons to complex II. Substrate-linked oxidative phosphorylation (OXPHOS), oligomycin-induced LEAK (LEAK), and non-coupled electron transfer (ET) is shown. **B/E)** Rates of H_2O_2 emission with non-limiting ADP expressed relative to protein content (pmol H_2O_2/µg protein/sec). FCCP, carbonyl cyanide p-(trifluoromethoxy) phenylhydrazone. Repeated measure ANOVA with with Sidak's multiple comparisons test was used to test for interaction and group effects. * denotes $p < 0.05$. Data are presented as mean ± SEM, n=4-7 per group.

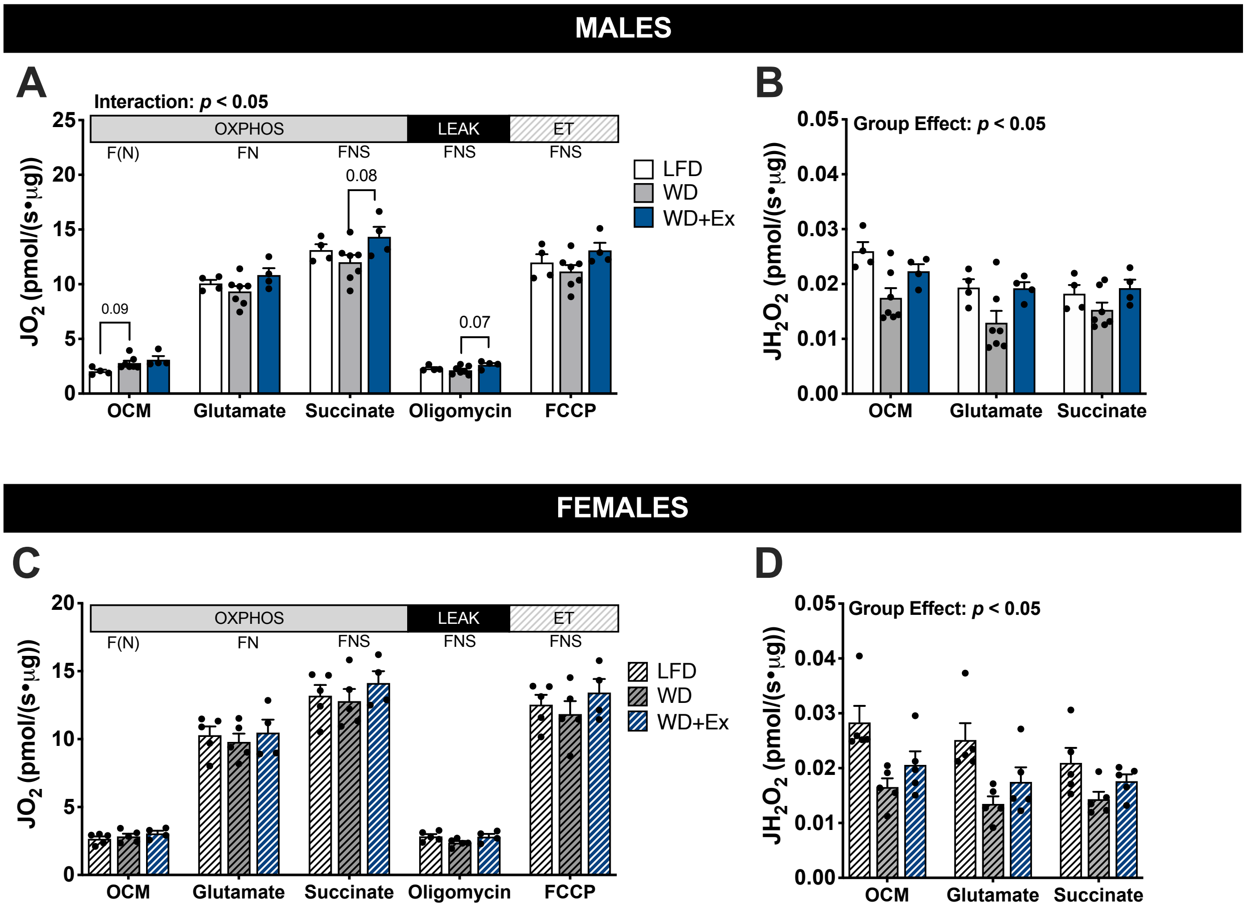

Supplementary Figure 2. Non-lipid supported skeletal muscle mitochondrial respiration. Quadricep muscles were collected after 4-h fast. A/D) Respiration of isolated mitochondria with non-limiting ADP. Rates of oxygen consumption (JO_2) are expressed relative to protein content (pmol/(s·µg)). Malate (M) and glutamate (G) are N-linked (N), donating electrons to complex I; succinate is S-linked (S), donating electrons to complex II. Substrate-linked oxidative phosphorylation (OXPHOS), oligomycin-induced LEAK (LEAK), and non-coupled electron transfer (ET) is shown. B/E) Rates of H_2O_2 emission with non-limiting ADP expressed relative to protein content (pmol H_2O_2/µg protein/sec). C/F) Electron leak to H_2O_2 calculated from simultaneous measurement of H_2O_2 emission and JO_2 during oxidative phosphorylation. FCCP, carbonyl cyanide p-(trifluoromethoxy) phenylhydrazone. Repeated measure ANOVA was used to test for effects of intervention and time. One-way Repeated measure ANOVA with Sidak's multiple comparisons test was used to test for interaction and group effects. * denotes $p < 0.05$. Data are presented as mean ± SEM, n=4-7 per group.

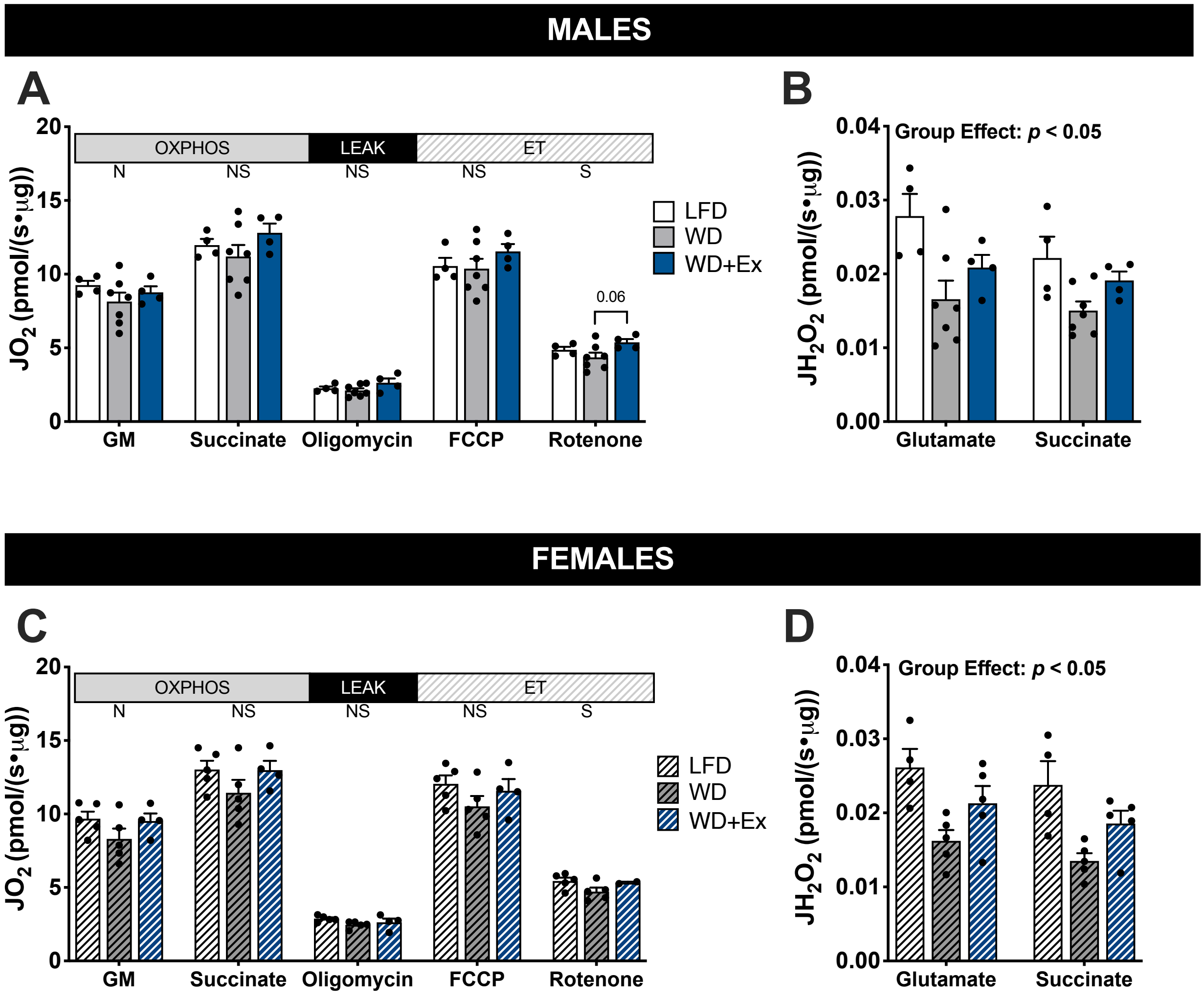

Supplementary Figure 3. Full representative blot images displayed in Figure 6. Boxed areas indicate regions displayed in manuscript. **A)** Full blot for OXPHOS (left) un-cut before imaging (imaged in 800 channel). Ponceau stain depicts full blot (right). **B)** Full blot for HADH (left) un-cut before imaging (imaged in 700 channel). Ponceau stain depicts full blot (right). **C)** Full blot for CPT1 (left) un-cut before imaging (imaged in 700 channel). Ponceau stain depicts full blot (right). **D)** Full blot for CD36 (left) un-cut before imaging (imaged in 700 channel). Ponceau stain depicts full blot (right). **E)** Full blot for PGC1α (left) cut below 75 kDa before imaging (imaged in 700 channel). Ponceau stain depicts full blot before cutting (right). **F)** Full blot for TFAM (left) un-cut before imaging (imaged in 700 channel). Ponceau stain depicts full blot (right).

A) OXPHOS

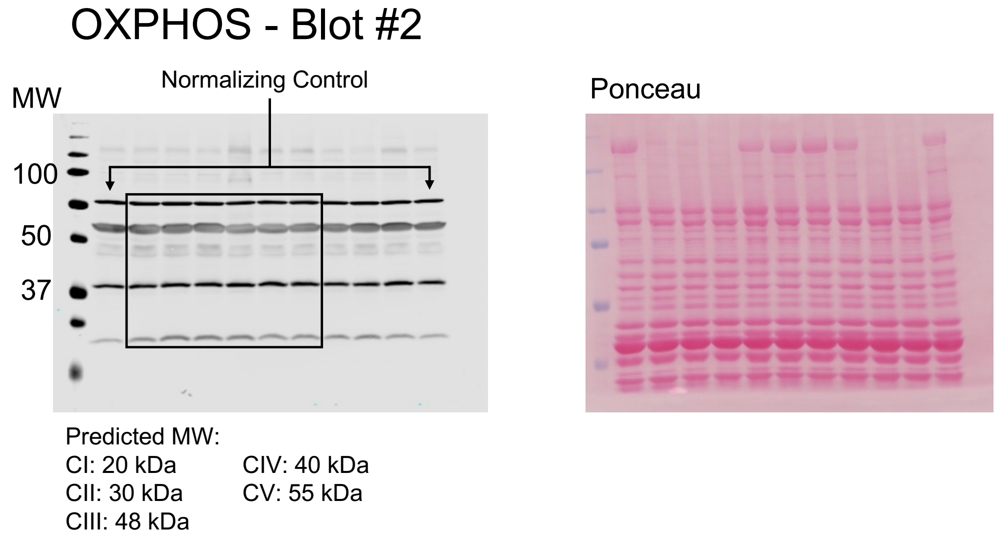

B) HADH

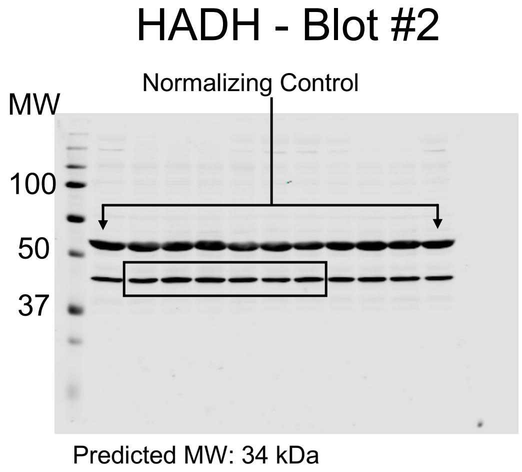

Ponceau for HADH is shown above in Supplementary Figure 3A.

C) CD36

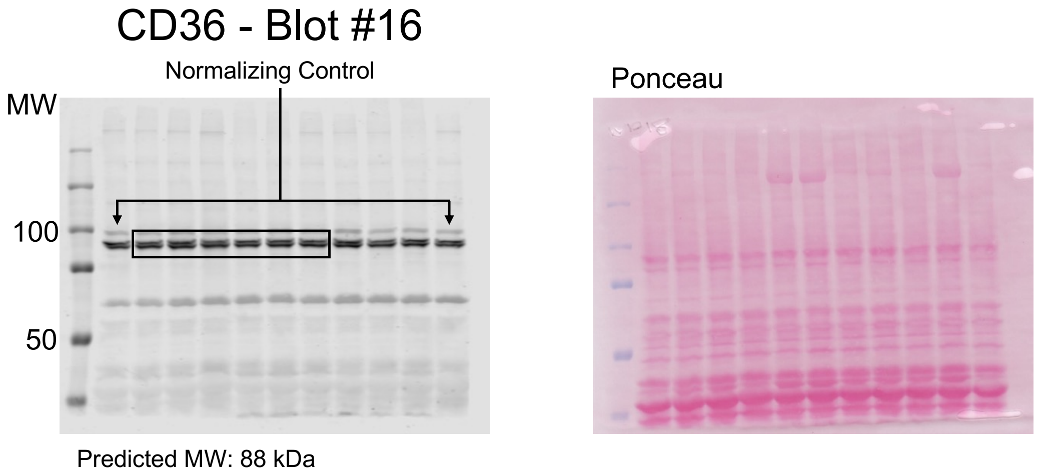

D) CPT1

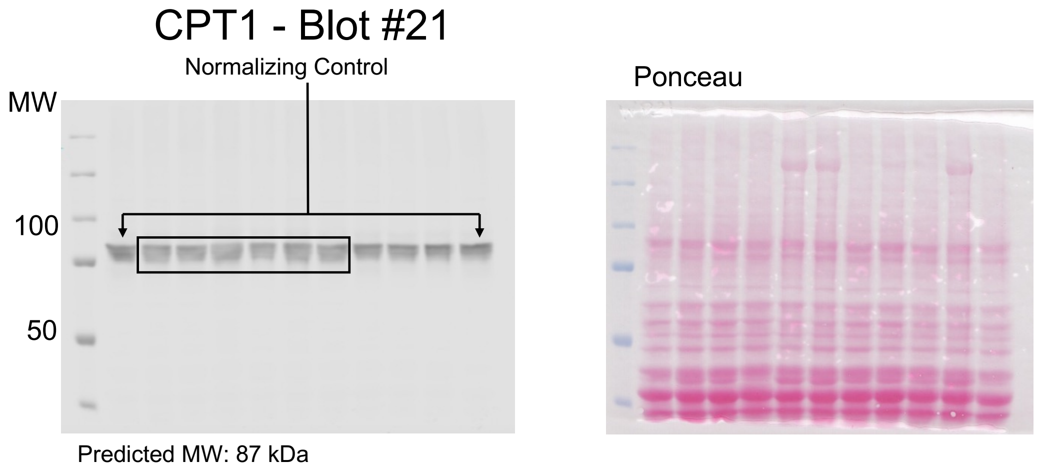

E) PGC1α

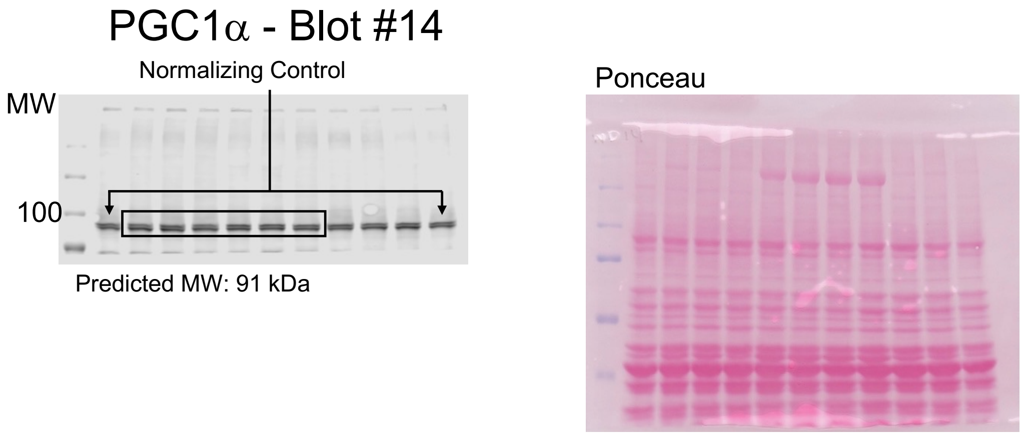

F) TFAM

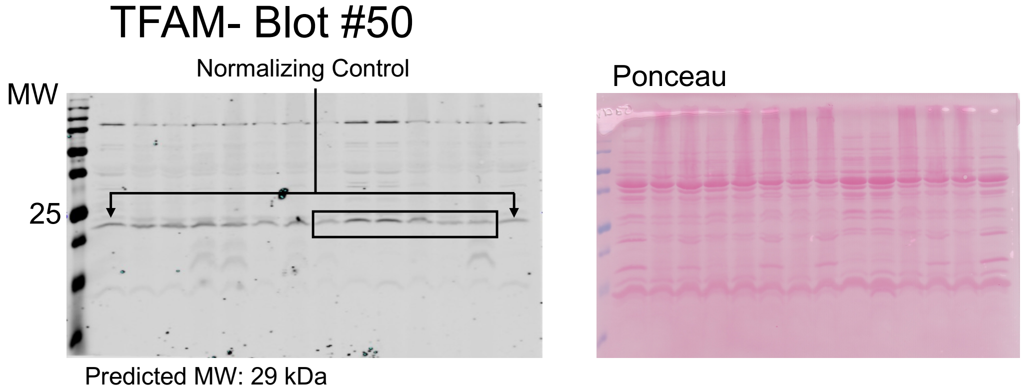

Supplementary Figure 4. Exercise training sessions were completed between 14:00-17:00 during the light cycle. During week 0, mice acclimated to the treadmill and exercise bouts began at 6 m/min at 0% incline for 5 minutes, then progressed to 9 m/min for 5 minutes and ended with 12 m/min for 5 minutes. In week 1, exercise bouts began at 12 m/min at 5% incline and progressed to 17 m/min for 45 min at 10% incline. During weeks 2, 3, and 4, mice exercised at 17 m/min for 45 min at 10% incline. Mice were encouraged to continue running with an air puff or mild shock plate at the back of the treadmill. Mice were removed from the treadmill when they refused to run, despite sitting on the shock grid for up to 5 seconds. Sedentary mice were not introduced to the treadmill but were routinely handled. Data are presented as mean ± SEM, n=3-5 per group.

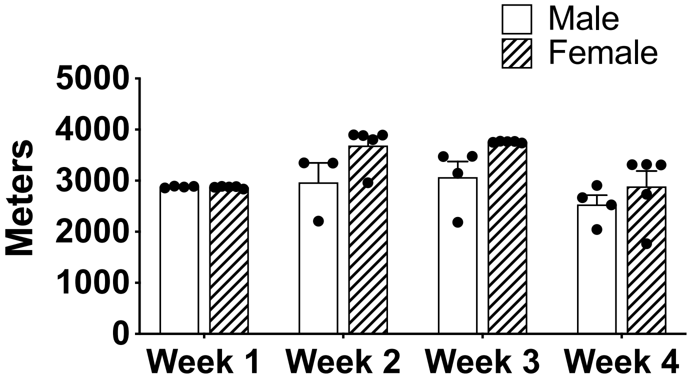

Supplement: Supplementary file 1 — Figures S1–S4 [file PHY2-10-e15543-s001.pdf]
